# Supplementary material for: Correlation between BAP1 Localization, Driver Mutations, and Patient Survival in Uveal Melanoma
Source: Cancers (Basel). 2022 Aug 25;14(17):4105. doi: 10.3390/cancers14174105 (PMC9454448; doi:10.3390/cancers14174105)
Supplement: Supplementary file 1 [file cancers-14-04105-s001.zip › cancers-1858458-supplementary.pdf]

Table S1. Primers for PCR amplification of FFPE derived tumor DNAs.

Amplicon	Forward Primer	Reverse Primer	Amplicon Length	Annealing Temp
BAP1 Exon 1_3	CGTTGTCTGTGTGTGGGAC	AGGCTGCTGCTTTCTGTGAG	566 bp	54°C
BAP1 Exon 1_2	GTGTGTGGGACTGAGGG	TGAGGAAAGGAAAGCAGTAG	368 bp	50°C
BAP1 Exon 3	CGTGGAAGATTTCGGTAAG	TTTACAACGTAGGGTTCCTG	281 bp	50°C
BAP1 Exon 4	ATGCTGATTGTCTTCTCCCC	CTCCATTTCCACTTCCCAAG	261 bp	54°C
BAP1 Exon 5	CTTGGGGCTTGCAGTGAG	ATGGGTAGCATTCCCAGTG	254 bp	53°C
BAP1 Exon 6_7	TTTGCCTTCCACCCATAGTC	AGCTCCCTAGGAGGTAGGC	412 bp	53°C
BAP1 Exon 6	CACCCATAGTCCTACCTGAG	ACTAAGGCCATTCTGCTTC	247 bp	50°C
BAP1 Exon 7	CTGTGGGAGCTGATGTG	TCCCTAGGAGGTAGGCAG	267 bp	50°C
BAP1 Exon 8	TGTCTTCCTTCCCACTCCTG	TCTGTCCCTCCCAAAGTAGG	218 bp	54°C
BAP1 Exon 9	GGATATCTGCCTCAACCTGATG	GAAGGGAGGAGGAATGCAG	256 bp	55°C
BAP1 Exon 10	TGAGCCCTGGATTCTGTTGT	CAGACATTAGCGGGTGGC	225 bp	55°C
BAP1 Exon 11	GGGGAGACTGTGAGCTTTTC	CATGGGAAAATTGCCTGTTG	325 bp	54°C
BAP1 Exon 12	GCTGTGAGTGTCTAGGCTCAG	AGACTGAGATATTCAGGATG GG	270 bp	52°C
BAP1 Exon13A	CTGCCTTGGATTGGTCTGAT	CAACACCATCAACGTCTTGG	567 bp	55°C
BAP1 Exon 13B	TGATGACAGGACCCAGATCA	GCTGTCAGAACTTGATGCCA	595 bp	55°C
BAP1 Exon 13_1	TGTATTTAAGCCATTCTGGG	AGAGGCCGTGTCTGTACTC	321 bp	50°C
BAP1 Exon 13_2	CTCAAAGAGTCCCAGAAGG	TATGCAGTCAACACGCAG	279 bp	50°C
BAP1 Exon 13_3	CTATCCGCTCAGCCAAC	GAGTGCAGGACACTTTGTG	288 bp	50°C
BAP1 Exon 14	CCAAGTGACCACAAAGTGTCC	AGCTCAGGCCTTACCCTCTG	275 bp	56°C
BAP1 Exon 15_16	CTAGCTGCCTATTGCTCGTG	GAGGGGAGCTGAAGGACAC	409 bp	54°C
BAP1 Exon 15	CATCCTTGCCTCTAGCTG	AGCCACCAGTGGACCTC	232 bp	54°C
BAP1 Exon 16	CACTGGTGGCTGCTCTG	GAGCTGAAGGACACGGC	194 bp	52°C
BAP1 Exon 17	CTGAGCACTATGGGGCTGAT	TCTTAACTGGAATGCCCTGC	496 bp	55°C
BAP1 Exon 17	CTTGAGCAGACCTTGGG	TCCAGCTGGGACTATTCAG	339 bp	54°C
EIF1AX Exon 1	CCTCCAGCACCTACTTGGTC	CCTGGGTGACCTGCAATCTA	215 bp	61°C

EIF1AX Exon 2	AAATGGTTTTATAAGCCTTAATT TCA	TAATCGTGCCACCACACTTC	298 bp	52°C
SF3B1 R625	AGAGTGGAAGGCCGAGAGAT	TACCAGTGTGTCTCGCTTGC	348 bp	52°C

Note: 20 or 50 ng of DNA was used for PCR reactions. PCR was performed with Platinum *Taq* DNA Polymerase PCR Kit (ca. no. 10966026, Thermo Fisher Scientific, MA) in a reaction volume of 25 μL. To increase the efficiency of DNA amplification in FFPE derived DNA, optimized conditions for a subset of primers required 1.5x and 2x the standard amount of $MgCl_2$ and *Taq* polymerase, respectively. Bolded sequences denote primers that were redesigned to amplify a shorter amplicon. The listed annealing temperatures were experimentally determined.

Table S2. Patient Cohort Characteristics. Information is based on available clinical details from all patients (n=100). Overall survival was calculated from the date of diagnosis to death or last follow-up. Patients were censored if no death occurred at the time of last follow-up.

Variable	No. (%)
Age, median (range), years	67.5 (23-96)
Sex	
Male	51 (51)
Female	47 (47)
Unknown	2 (2)
TNM staging	
**I-IIB**	51 (51)
I	8 (8)
IIA	15 (15)
IIB	28 (28)
**IIIA-IIIC**	47 (47)
IIIA	29 (29)
IIIB	14 (14)
IIIC	4 (4)
Unknown	2 (2)
Chromosome 3 status	
Disomy	27 (27)
Monosomy	37 (37)
Polysomy	4 (4)
Unknown	32 (32)
Chromosome 8q status	
Normal	19 (19)
Gain	51 (51)
Unknown	30 (30)
Metastasis	
Yes	39 (39)
No	35 (35)
Unknown	26 (26)
Time to metastasis, median (range), months	20.2 (0-74.5)
Follow-up interval, median (range), months	38.0 (0-88.8)

Table S4. Summary of UMs with nBAP1 Loss and the Presence of cBAP1 in the Context of Class of Driver Mutations and Chromosomal Alterations.

	Total	25% or more nBAP1 loss	Percent nBAP1 loss	> 10% cBAP1	Percent cBAP1 with nBAP1 loss
BAP1 Truncating	15	15	100	9	60

BAP1 Mis-sense	10	8	80	8	100
BAP1 Splicing	1	1	100	0	0
Total BAP1 mutations	26	24	92	17	71
No sequencing results	22	10	45	9	90
Disomy 3	20	4	20	3	75
Monosomy 3	37	30	81	20	67
Chr 3 gain	2	1	50	1	100
EIF1AX mutation	6	1	17	0	0
SF3B1 mutation	9	1	11	1	100
TOTAL stained	96	55	57	30	55

Table S5. Prediction of BAP1 Gene Mutation and Chromosome 3 Aneuploidy using Predominant BAP1 Staining Pattern. 2x2 table created based on the BAP1 IHC and Chr3 status (n = 73).

		Chr3 and *BAP1* Gene Status		
		**Chr3 Disomy + *BAP1* Wildtype**	**Chr3 Aneuploidy or *BAP1* Mutation (any)**	**Total**
**BAP1 IHC**	Retained	20	6	26
	Loss	5	41	46
	Total	25	47	72

Sensitivity: 87.2% (41/47); Specificity: 80% (20/25); Positive Predictive Value (PPV): 89.1% (41/46); Negative Predictive Value (NPV): 76.9% (20/26).

Table S6. Levels of BAP1 mRNA and BAP1 mutation in TCGA UM Cohort.

**TCGA Samples**	**BAP1 mRNA level**	**Mets.**	**Exon**			**Mutation type**	**Predicted NMD**
A985	3.33	N	exon14	c.1827_1855del	p.S609fs	truncating	Yes
A8KN	3.67	N	exon13	c.1358_1359del	p.K453fs	truncating	Partial
A9EU	3.76	N	exon13	c.C1321T	p.Q441X	truncating	Partial
A8KK	4.09	N	exon5	c.371delC	p.P124fs	truncating	Partial
AA8T	4.10	N	exon13	c.1491dupT	p.E498_I499delinsX	truncating	No or partial
A984	4.38	Y	exon13	c.1262delA	p.K421fs	truncating	Partial
A9EF	4.50	N	exon9	c.C669G	p.Y223X	truncating	Cryptic splice in exon
A9EV	4.77	Y	exon9	c.689_729del	p.L230fs	truncating	NMD
A9EE	4.92	Y	exon6	c.434_435insTGAA GCTTTGCCTT	p.A145fs	truncating	Exon skipping; no NMD
A980	5.55	N	exon11	c.1013delC	p.P338fs	truncating	Mutant allele not detected
A9F1	5.93	N	exon3	c.C99G	p.Y33X	truncating	No
AA9A	5.97	Y	exon7	c.G553C	p.G185R	missense	NA
AA8N	6.24	N	exon4	c.A233G	p.N78S	missense	NA
A888	6.28	N	exon7	c.A518G	p.Y173C	missense	NA
A9EX	6.34	Y	exon3	c.C118T	p.Q40X	truncating	No
A9EI	6.39	Y	exon16	c.2015_2024del	p.D672fs	truncating	No
A9EO	6.69	N	exon16	c.A2054T	p.E685V	missense	No
A8KL	6.80	N	exon3	c.79dupG	p.V27fs	truncating	No
A88A	6.86	Y	exon4	c.166_197del	p.R56fs	truncating	No
A9F8	7.10	N	exon4	c.A203G	p.D68G	missense	NA

UM data from uvm_tcga_pan_can_atlas_2018 [1] are shown. Tumor IDs, whether it had undergone metastasis, the BAP1 mutation and type, the exon the mutation resided in, the level of BAP1 mRNA and whether the mRNA had undergone NMD are shown in separate columns. Mets (Metastasis).

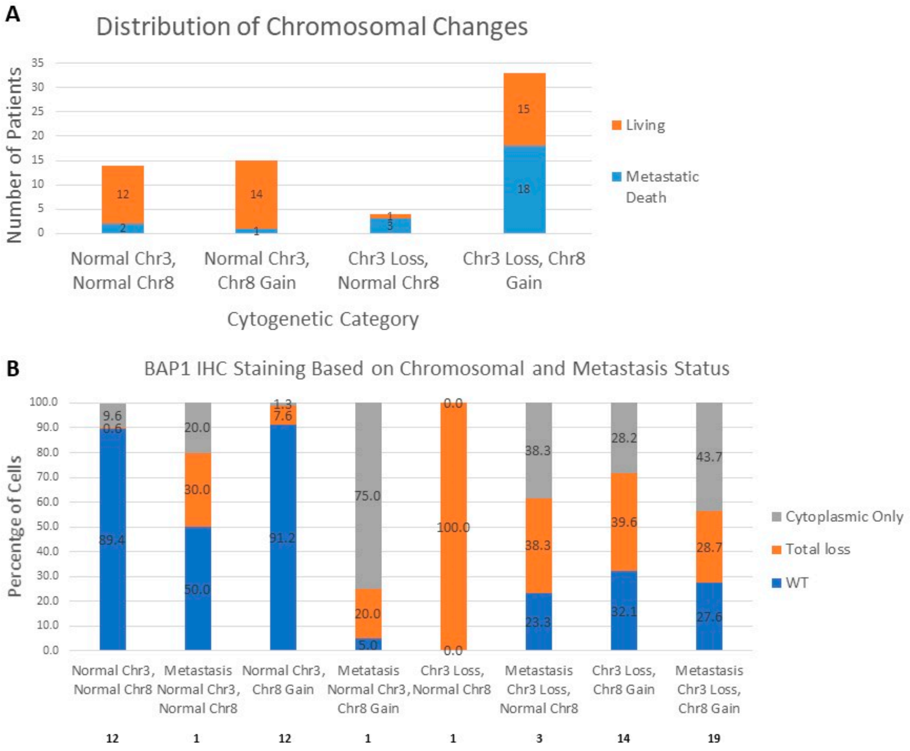

Figure S1. Chromosomal Status of UM Tumors and Prognosis. The ploidy of chromosome 3 and 8q were obtained from clinical FISH performed on biopsy/resected tissues. (A) Tumors are classified based on status of chromosome 3 (Disomy 3 or LOH3) and 8q (Disomy 8q or 8q gain). The color of the stacked bar indicates the number of patients who died due to metastasis (blue) or who are currently alive (orange). Only patients whose tumors had both cytogenetic information were included in this figure (n = 66). Tumors with polyploid status or gain of chr3 are not included. (B) Immunohistochemistry staining pattern of tumors are graphed based on chromosomal and metastatic status (n= 63). Percentage of cells with WT BAP1 (blue), total loss of BAP1 (orange), and cytoplasmic only cBAP1 expression (grey) are indicated. The number of tumors in each category is denoted below the title of each stacked column.

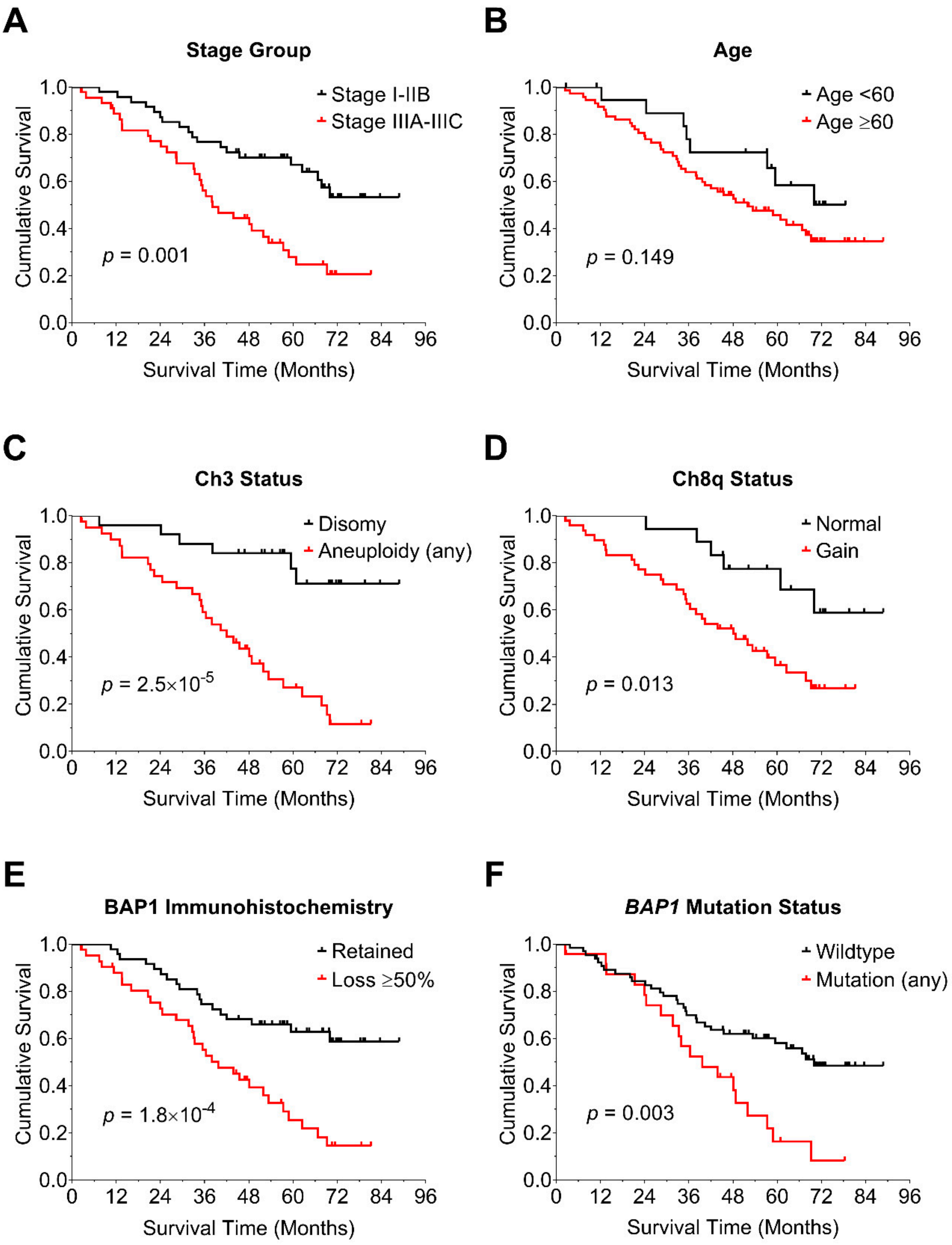

Figure S2. Survival Analysis According to Molecular Characteristics. Kaplan-Meier estimates of overall survival in patients according to (A) Stage, (B) Age, (C) Chromosome 3 status, (D) Chromosome 8q status (E) BAP1 immunohistochemistry where loss corresponded to loss of nBAP1 as the predominant staining pattern (n = 90) (F) BAP1 mutation. Mantel Cox log-rank tests were performed to determine statistical significance ($P < 0.05$).

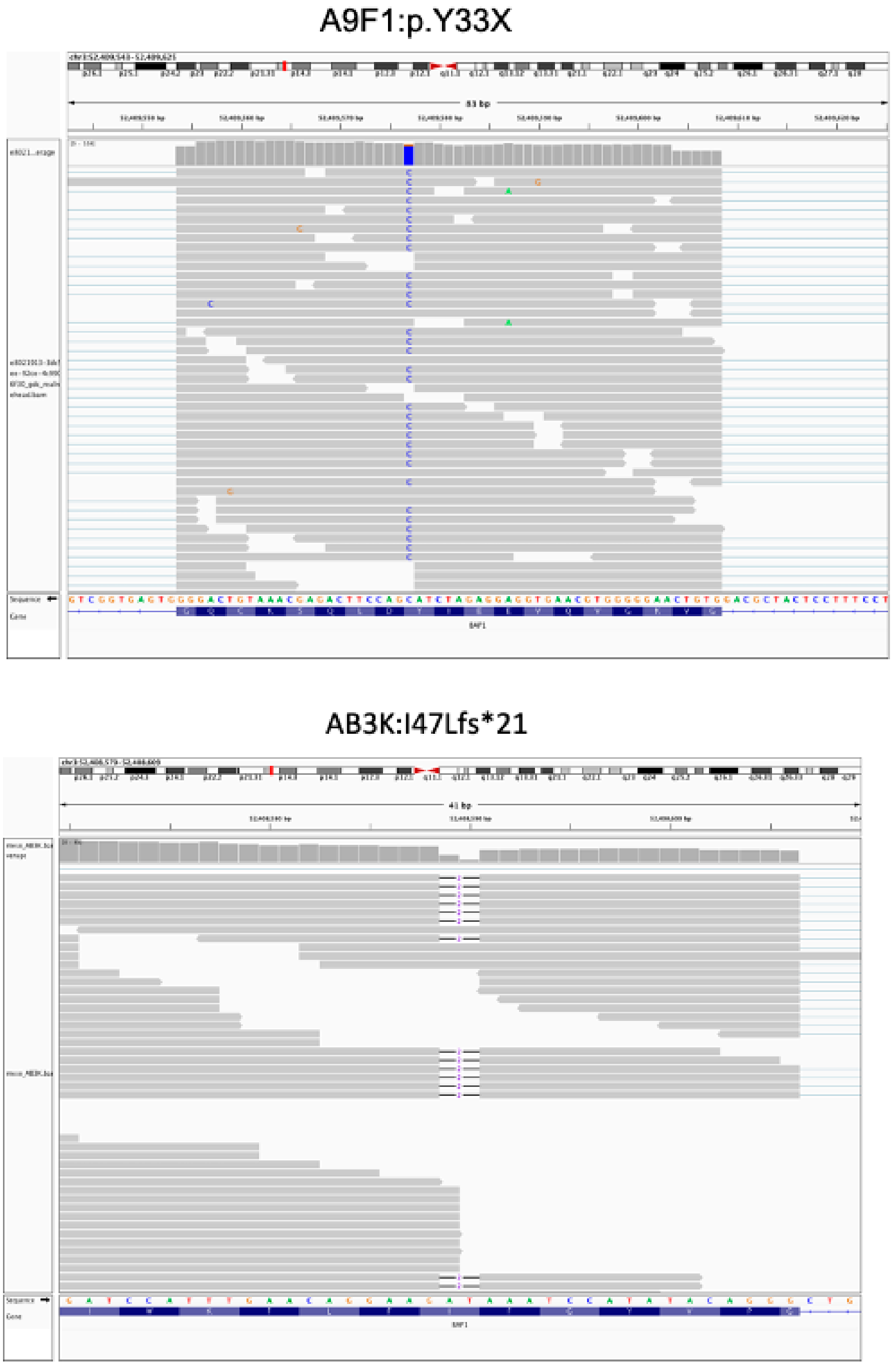

Figure S3. Examples of Tumors with Truncating Mutations in BAP1 that have escaped NMD. 1. UM tumor A9F1 with truncating BAP1 mutation p.Y33X that has undergone escape from NMD. 2. Pleural mesothelioma tumor AB3K with frameshift mutation leading to I47Lfs*21. Visualization of BAP1 transcripts was performed with the Integrative Genomics Viewer (IGV).

References:

1. Robertson, A.G.; Shih, J.; Yau, C.; Gibb, E.A.; Oba, J.; Mungall, K.L.; Hess, J.M.; Uzunangelov, V.; Walter, V.; Danilova, L.; *et al.* Integrative analysis identifies four molecular and clinical subsets in uveal melanoma. *Cancer Cell* **2018**, *33*, 151.
